# The effect of sucralose on flavor sweetness in electronic cigarettes varies between delivery devices

**DOI:** 10.1371/journal.pone.0185334

**Published:** 2017-10-02

**Authors:** Kathryn Rosbrook, Hanno C. Erythropel, Tamara M. DeWinter, Mark Falinski, Stephanie O’Malley, Suchitra Krishnan-Sarin, Paul T. Anastas, Julie B. Zimmerman, Barry G. Green

**Affiliations:** 1 The John B. Pierce Laboratory, New Haven, Connecticut, United States of America; 2 Center for Green Chemistry & Green Engineering at Yale University, New Haven, Connecticut, United States of America; 3 Department of Chemical and Environmental Engineering, Yale University, New Haven, Connecticut, United States of America; 4 Department of Forestry and Environmental Studies, Yale University, New Haven, Connecticut, United States of America; 5 Department of Psychiatry, Yale School of Medicine, New Haven, Connecticut, United States of America; 6 Department of Surgery (Otolaryngology), Yale School of Medicine, New Haven, Connecticut, United States of America; Louisiana State University, UNITED STATES

## Abstract

The appeal of sweet electronic cigarette flavors makes it important to identify the chemical compounds that contribute to their sweetness. While volatile chemicals that produce sweet aromas have been identified in e-liquids, there are no published reports of sugars or artificial sweeteners in commercial e-liquids. However, the sweetener sucralose is marketed as an e-liquid additive to commercial flavors. The primary aims of the study were to determine if sucralose is delivered in sufficient concentration in the inhaled aerosol to enhance flavor sweetness, and whether the amount delivered depends on the e-liquid delivery system. Thirty-two adult smokers rated flavor intensity, sweetness, harshness and liking/disliking for 4 commercial flavors with and without sucralose (1%) using 2 e-cigarette delivery systems (cartridge and tank). Participants alternately vaped normally or with the nose pinched closed to block perception of volatile flavor components via olfaction. LC/MS was used to measure the concentration of sucralose in the e-liquid aerosols using a device that mimicked vaping. Sweetness and flavor intensity were perceived much more strongly when olfaction was permitted. The contribution of sucralose to sweetness was significant only for the cartridge system, and the chemical analysis showed that the concentration of sucralose in the aerosol was higher when the cartridge was used. Together these findings indicate that future regulation of sweet flavor additives should focus first on the volatile constituents of e-liquids with the recognition that artificial sweeteners may also contribute to flavor sweetness depending upon e-cigarette design.

## Introduction

With the goal of improving public health, the Food and Drug Administration has the authority to evaluate and regulate factors such as the design and constituents of tobacco products that contribute to the appeal of the product. A significant factor in the appeal of electronic cigarettes (e-cigarettes) is the availability of a wide variety of flavors [1–3]. Surveys have shown that sweet and fruity flavors are among the most popular, particularly with youth and young adults [4–8], and a recent laboratory study confirmed there is a significant association between sweetness and the degree of liking of e-cigarette flavors [9]. Sweet flavors may therefore heighten the risk of nicotine exposure in this young, at-risk cohort [10]. There is also concern that some sweet flavorants commonly used in foods and beverages have irritant properties when inhaled [11–13]. It is therefore important to identify the chemical compounds in commercial flavors (e-liquids) that contribute to the perceived sweetness of the inhaled aerosols and whether device characteristics can influence the delivery of these compounds.

In foods and beverages, sweetness arises primarily from the sense of taste and is served by specialized chemoreceptors in the tongue and palate that are sensitive to sugars and artificial sweeteners [14, 15]. However, odors can also have sweet perceptual qualities [16, 17], and the senses of olfaction and taste interact to produce overall flavor sweetness [18–20]. This perceptual interaction occurs when volatile molecules that are released in the mouth reach the olfactory epithelium via the nasal pharynx (“retronasal olfaction”) and produce odors that are perceived in the mouth rather than in the nose [21–24]. Perceptual “referral” of retronasal olfaction from the nose to the mouth binds tastes and odors into integral perceptions of flavor. Accordingly, it can be difficult to know whether the sweetness of a flavor arises from sweet-tasting non-volatile ingredients, sweet-smelling volatile ingredients, or both.

Analyses of the constituents of commercially available flavors have identified several well-known volatile chemicals that have sweet and/or fruity odors [25, 26] which we would be expected to be sensed via retronasal olfaction. However, the role of retronasal olfaction in the perception of e-cigarette flavors, including their sweetness, has never been studied. It is less clear whether non-volatile sweeteners that act through the sense of taste might also play a role. We have found no published reports of sugars or artificial sweeteners in chemical analyses of commercial e-liquids. The artificial sweetener sucralose is marketed as an e-cigarette flavor additive and is readily available on the internet. As a high potency sweetener, sucralose is an attractive potential additive, since in theory only very small quantities would be necessary to produce perceptible sweetness. Like sugars and other artificial sweeteners, sucralose is not a volatile molecule and thus is unlikely to be present in large quantities in inhaled e-cigarette aerosols.

The primary objective of the present study was therefore to determine if sucralose is an effective sweetening agent compared to the volatile constituents of sweet and fruity e-liquid flavors. Additionally, because preliminary psychophysical testing indicated that the effect of sucralose on sweetness seemed to depend on the type of e-cigarette used, we tested 2 different e-liquid delivery systems, a cartridge and a tank. To address both questions we employed a psychophysical procedure that enabled separation of the taste and retronasal olfactory components of flavor perception, together with analytical chemistry to quantify the amount of sucralose that was delivered in the e-cigarette aerosols.

## Materials and methods

### Psychophysical measurements

#### Subjects

Thirty-two adult smokers (16 females, 16 males) between 18–45 years of age, recruited through online advertisements and flyers posted around the Yale University campus and New Haven, CT, were paid to participate in the experiment. The research was conducted in accordance with the principles expressed in the Declaration of Helsinki, and the research protocol was approved by the Human Investigations Committee of the Yale University IRB, and all subjects gave written consent before participation. Prior to enrollment, all subjects were screened over the phone to determine eligibility. Only those who spoke English fluently and reported being current daily cigarette smokers for at least 1 year, in overall good health, not pregnant, and with no deficits of taste or smell were scheduled to participate. Subjects were asked to abstain from smoking for at least 10 hours before each session, which was confirmed by alveolar carbon monoxide levels <10 ppm[27] using the MicroCO breath carbon monoxide monitor (Micro Direct, Inc., Lewiston, ME). If the subject’s carbon monoxide reading was ≥10 ppm, the subject was rescheduled for another day.

#### Equipment and stimuli

**Fig 1:**
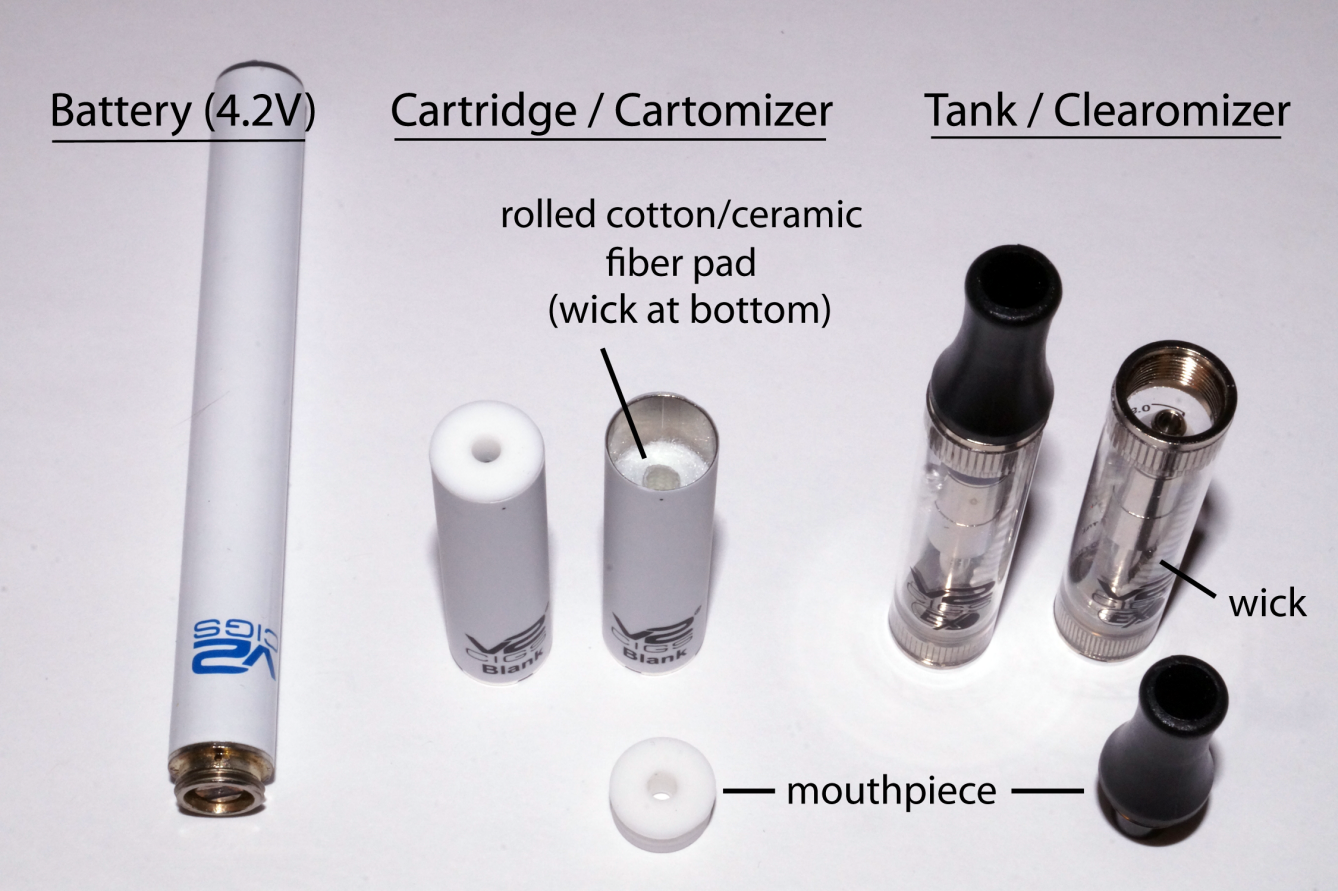
Shown are the 2 e-cigarette delivery systems that were tested. The systems (cartridge/cartomizer and tank/clearomizer) are powered by the same battery (left), but differ in the way e-liquids are stored and delivered to the heating element (i.e., in a saturated, rolled cotton/ceramic pad vs. a tank containing a braided cotton/ceramic wick) and in the design and size of the mouthpiece.

Fig 1 shows the 2 e-cigarette delivery systems were used: the V2 blank cartridge (cartomizer) atomizer and the V2 EX blank tank (clearomizer) atomizer (V2^™^, VMR Products LLC, Miami, FL). Both delivery systems were powered by the standard 79mm V2 e-cigarette batteries (4.2V). Four commercial e-liquid flavors were purchased from an online retailer (AmericaneLiquidStore^™^, Wauwatosa, WI): Strawberry, Vanilla, Watermelon and Cherry. All e-liquids nominally contained 12 mg/mL of nicotine in a 50/50 PG/VG base. However, gas chromatography (GC) determined the actual nicotine contents were 13.3, 14.1, 13.3 and 14.7mg/mL, and analysis by liquid chromatography followed by detection by refractive index (LC/RI) showed the actual PG/VG ratios were 55/45, 80/20, 60/40, and 49/51, respectively.

The e-liquid stimuli were prepared both with and without added sucralose (Sigma-Aldrich; St. Louis, MO) in a concentration of 1% (wt/vol). Use of this concentration was based on pilot testing which indicated that 1% was sufficient to evoke a weak sweet taste when the e-liquids were vaped using the V2 cartridge with the nose pinched closed, and tests with 5% sucralose did not appear to significantly increase sweetness. The blank cartridges and tanks were filled with 500 μL of e-liquid on the day of testing at least 1h prior to the testing session. The functionality of each cartridge was tested after filling by using a syringe to pull air through the e-cigarette to simulate inhalation. On the rare occasion that a cartridge or tank failed (i.e., no vapor was visible inside the syringe) the cartridge was discarded and a new one was filled and tested. Tanks and cartridges were not used across participants and were disposed of upon test completion.

#### Experimental design

A within-subjects design was used in which all subjects served in every condition of the experiment. To assess the effects of retronasal olfaction and sucralose on flavor, the design included 2 olfaction and 2 sucralose conditions. The olfaction conditions were nose-open (Olfaction), which allowed the inhaled aerosol to reach the olfactory epithelium through the oro-nasal pharynx during exhalation (i.e., via retronasal olfaction), and nose-closed (No-Olfaction), which prevented the inhaled aerosol from reaching the olfactory epithelium. The 2 sucralose conditions were the e-liquid with sucralose added (Sucralose) and the e-liquid without sucralose added (No-Sucralose). The olfactory and sucralose conditions were paired in a crossed design to yield 4 test conditions: (1) Olfaction + Sucralose, (2) Olfaction + No-Sucralose, (3) No-Olfaction + Sucralose, and (4) No-Olfaction + No-Sucralose.

To investigate the potential effects of e-cigarette design on flavor delivery and perception, the 4 commercial flavors (E-liquids) were sampled under all 4 testing conditions using both the V2 cartridge and V2 EX tank (Delivery Systems). The 2 systems were tested in separate sessions and replicate ratings were collected in 2 additional sessions. Subjects therefore served in a total of 4 sessions with each session containing 16 trials (4 test conditions x 4 e-liquids). On each trial data were collected on sensation intensity *(overall flavor*, *sweetness*, and *harshness/irritation*) and *liking/disliking*. Within each session the Sucralose and No-Sucralose conditions were blocked to limit potential carryover of sucralose sweetness into No-Sucralose trials. The order in which the sucralose conditions were tested was counterbalanced across subjects and the e-liquids were presented in 4 different pseudo-random orders.

#### Testing procedure and subjective ratings

In the first testing session subjects were read instructions about how to use the generalized version of the Labeled Magnitude Scale (gLMS)[28–30] to rate sensation intensity and the Labeled Hedonic Scale (LHS)[31] to rate liking/disliking. The gLMS and LHS scales were presented sequentially on a computer monitor and the subjects made their ratings by using a mouse to move a cursor to the appropriate locations on the scale. Subjects were given practice using both scales by rating the perceived intensities and liking/disliking of 15 remembered or imagined sensations that were read to them by the experimenter.

Instructions and practice were also given in the use of the 2 e-cigarette delivery systems. In order to fully activate the heating element in V2 e-cigarettes, on each trial subjects took two priming puffs into the mouth only, exhaling them both through the mouth, before fully inhaling a third puff into the airways, which was also exhaled through the mouth. (Note that with the nose open, expired volatiles can nevertheless reach the olfactory epithelium via the retronasal route, including during normal breathing immediately following exhalation). This vaping procedure was used throughout testing. On No-Olfaction trials subjects wore a nose clip which remained on the nose as they vaped and while making ratings of sensation intensity and liking/disliking. The clip was removed between trials to allow normal breathing through the nose.

On each trial the **s**ubject was given a number coded e-cigarette that gave no indication of its flavor, sucralose content, or nicotine concentration. The subjects were instructed to vape each puff as they had during training: 2 priming puffs into the mouth before fully inhaling the third puff, which was always exhaled through the mouth. Subjects rated sensation intensity and liking/disliking immediately after exhalation. The instructions were to base their ratings exclusively on their perception of the third (inhaled) puff. During a 5-min break after the first 8 trials subjects rinsed the mouth with deionized (DI) water to remove any lingering taste before beginning the next block.

#### Statistical analysis

Analyses were carried out using the repeated-measures module of the advanced linear modeling tool of Statistica^™^ 13. An overall ANOVA was conducted first with Flavor Characteristic as the dependent variable with 3 levels (*overall intensity*, *sweetness*, and *harshness/irritation*) to evaluate the predicted effects of Sucralose, Olfaction and Delivery System on flavor perception. Specifically, it was expected that ratings of *overall flavor* and *sweetness* would be higher in the Olfaction and Sucralose conditions, and that the effect of adding sucralose would be dependent upon Delivery System. After finding significant main effects and interactions among these factors as well as E-liquid, individual repeated-measures ANOVAs together with Tukey HSD tests were conducted to clarify the sources of the interactions. Ratings of Liking/Disliking were analyzed in a separate ANOVA. Prior to statistical analysis the raw perceived intensity ratings were converted to log_10_ to adjust for the log-normal distribution of the data that is characteristic of the gLMS [29, 32]. No log conversion is necessary for *liking/disliking* data collected using the LHS[31].

### Analytical chemistry methods

An analytical system was setup that mimicked the parameters of vaping.

The vaping setup consisted of a micro diaphragm pump (KNF Neuberger Inc., Trenton, NJ) powered by a DC power supply (Velleman, Inc., Fort Worth, TX) and regulated with a needle valve, followed by a flow meter (Omega Engineering, Inc., Norwalk, CT), a cold finger trap chilled in liquid nitrogen (Airgas, Radnor, PA), and a reducing connector (McMaster-Carr, Elmhurst, IL) attached directly to the cold-finger trap. Before filling the cartridge or tank with e-liquid, its resistance was measured using an electronic multimeter. Subsequently, approximately 500 mg or 700–800 μL of the e-liquids in question were added to the cartridges and tanks, respectively, and these were allowed to settle for at least 1 h. To carry out the vaping experiment, a flow rate of 1.1 L/min was set using a blank cartridge and the filled cartridge or tank was subsequently attached to the reducing connector and the V2 standard 79mm battery was screwed onto the cartridge or tank. All connections were sealed using threaded seal tape (McMaster-Carr, Elhurst, IL) where necessary, or parafilm (Bemis, Neenah, WI). The power supply was controlled using a programmable Arduino Uno controller (Arduino, Somerville, MA) which was programmed to take 3 primer puffs of 0.25 s followed by one, 4-s puff. This procedure resulted in a puff volume of 73.3 ml and was based on preliminary in-house vaping topography data that were collected from 11 subjects who reported regular e-cigarette use as well as data from the literature [33]. After allowing the e-cigarette to cool down for 30 s, the procedure was repeated for a total of 20 times to collect a sufficient amount of vapor. Once 20 puffs were collected, the setup was disassembled and the cold finger trap was allowed to thaw. The content of the trap was dissolved in DI water and the condensed liquid inside the removable mouthpiece of the cartridge or tank was carefully rinsed into a vial.

#### Liquid chromatography—Mass spectrometry (LC/MS)

The amount of sucralose in both the trap and the mouthpiece was quantified using LC/MS (Varian 500-MS, 212-LC pump system, electrospray ionization, Palo Alto, CA) based on a method established previously[34]. A reverse phase column (Atlantis dc18; Waters Limited, Milford, MA) with 100Å particle pore size, 3 μm particle size, 3.9 mm ID, 150 mm length, and the corresponding inline guard column (Atlantis dc18 VanGuard cartridge; Waters Limited Milford, MA) was used. Sample injection was done with a Varian Prostar 420 autosampler (Palo Alto, CA) into a 20 μL sample loop. HPLC-grade water (solvent A) and acetonitrile (solvent B, both Fisher Scientific, Waltham, MA) were used to run an elution gradient at 35°C and a flow rate of 200 μL/min for a total runtime of 46 min. B was held constant at 5% for 2 min followed by a linear gradient to reach 95% at 15 min, and held constant again until 30 min. To ensure complete removal of any material, a steep gradient to 5% B was run until 31 min, and B was held again at 5% until 46min. The MS was run in negative mode with a capillary voltage of 65V, RF loading of 110%, and needle voltage of -4000V.

## Results

### Psychophysical data

**Fig 2:**
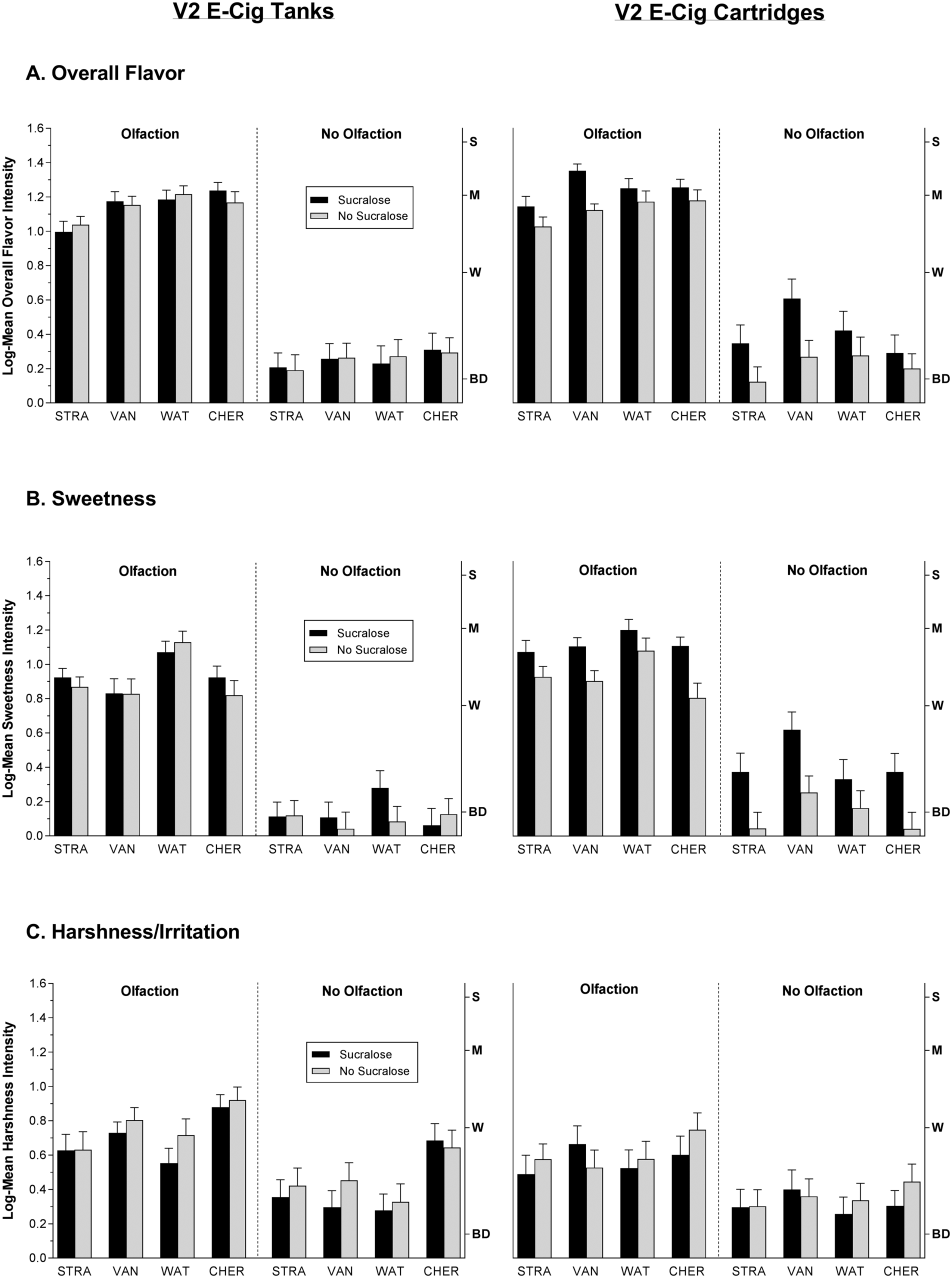
Ratings of overall flavor, sweetness and harshness intensity. Log_10_ mean ratings of (A) Overall Flavor, (B) Sweetness and (C) Harshness/Irritation intensity for each e-liquid alone (gray bars) and with 1% sucralose added (black bars). Data from the V2 EX tanks are on the left and the V2 cartridges on the right under 2 vaping conditions: Olfaction = nose open, No Olfaction = nose closed. The data show that in the No Olfaction condition *overall flavor* and *sweetness* were significantly attenuated compared to the Olfaction condition for both E-cigarettes, and that sucralose produced significant increases in the same 2 flavor categories only when added to the V2 cartridges. The letters on the right y-axis represent semantic labels of intensity on the general Labeled Magnitude Scale (gLMS): BD = Barely Detectable; W = Weak; M = Moderate; S = Strong. Vertical bars are standard errors of the mean.

The data on the perceived intensity of the e-liquid flavors are summarized in Fig 2. Most evident in the figure is a strong dependence of *overall flavor* and *sweetness* intensity on the availability of retronasal olfaction (Olfaction condition). Analysis of the full data set confirmed there was a significant main effect of Olfaction (p<0.00001) and a 3-way interaction among Olfaction, Flavor Characteristic and e-Liquid [p<0.01]. For all 4 e-liquids, *overall flavor* and *sweetness* were rated slightly above “barely detectable” with the nose closed, whereas ratings of *harshness/irritation* were reduced to a lesser degree, particularly for the cherry-flavored e-liquid. The same analysis also showed that flavor perception depended upon Delivery System in complex ways, including a 4-way interaction with Sucralose, E-liquid, and Flavor Characteristic (p<0.01). To investigate this interaction separate ANOVAs were conducted on the data from the cartridge and tanks systems alone. Analysis of the cartridge data confirmed there was a main effect of Sucralose (p<0.0001) and an interaction between Sucralose and Flavor Characteristic (p<0.00001), which indicated that adding sucralose increased *overall flavor* and *sweetness* but not *harshness/irritation*. This advantage can be seen in Fig 2, where sucralose led to higher mean ratings of *overall flavor* and *sweetness* for all 4 e-liquids when the V2 cartridge (cartomizer) was used. The same analysis showed that an interaction between Sucralose and E-liquid fell just short of significance. In contrast, analysis of the V2 tank data found no main effect of Sucralose but instead uncovered an interaction between Sucralose and Flavor Characteristic (p<0.05) which reflected a slight decrease in *harshness/irritation* rather than an increase in *overall flavor* and *sweetness*.

To focus on the effect of adding sucralose under conditions of normal vaping with the nose open we conducted an ANOVA on the data from the Olfaction condition alone. The analysis revealed a significant 3-way interaction among Sucralose, Delivery System, and Flavor Characteristic (p<0.05) which was again driven by increased ratings of *overall flavor* and *sweetness* when the cartridge delivery system was used. The analysis did not find a significant 4-way interaction that included E-liquid (p = 0.16), confirming that the flavor enhancing effect of sucralose was similar across e-liquid flavors. However, an ANOVA on the *sweetness* data alone confirmed there was a significantly stronger effect of sucralose for the cartridge compared to the tank (Sucralose x Delivery System; p<0.001) which, however, was not consistent across e-liquids (Sucralose x E-liquid interaction; p<0.05). Tukey tests revealed that the latter interaction was driven by a significant increase in the sweetness of the cherry flavored e-liquid; increases in the sweetness of the other e-liquids were not significant. Analysis of the *harshness/irritation* data in the Olfaction condition uncovered a main effect of Delivery System (p<0.05), with the tank system producing more sensory irritation than the cartridge system. A 3-way interaction among Delivery System, Sucralose, and E-liquid (p<0.02) indicated a tendency toward a moderating effect of sucralose on *harshness/irritation* was not consistent across delivery systems or flavors, and none of the reductions in *harshness/irritation* of specific e-liquids was statistically significant (Tukey HSD tests, p>0.05).

**Fig 3:**
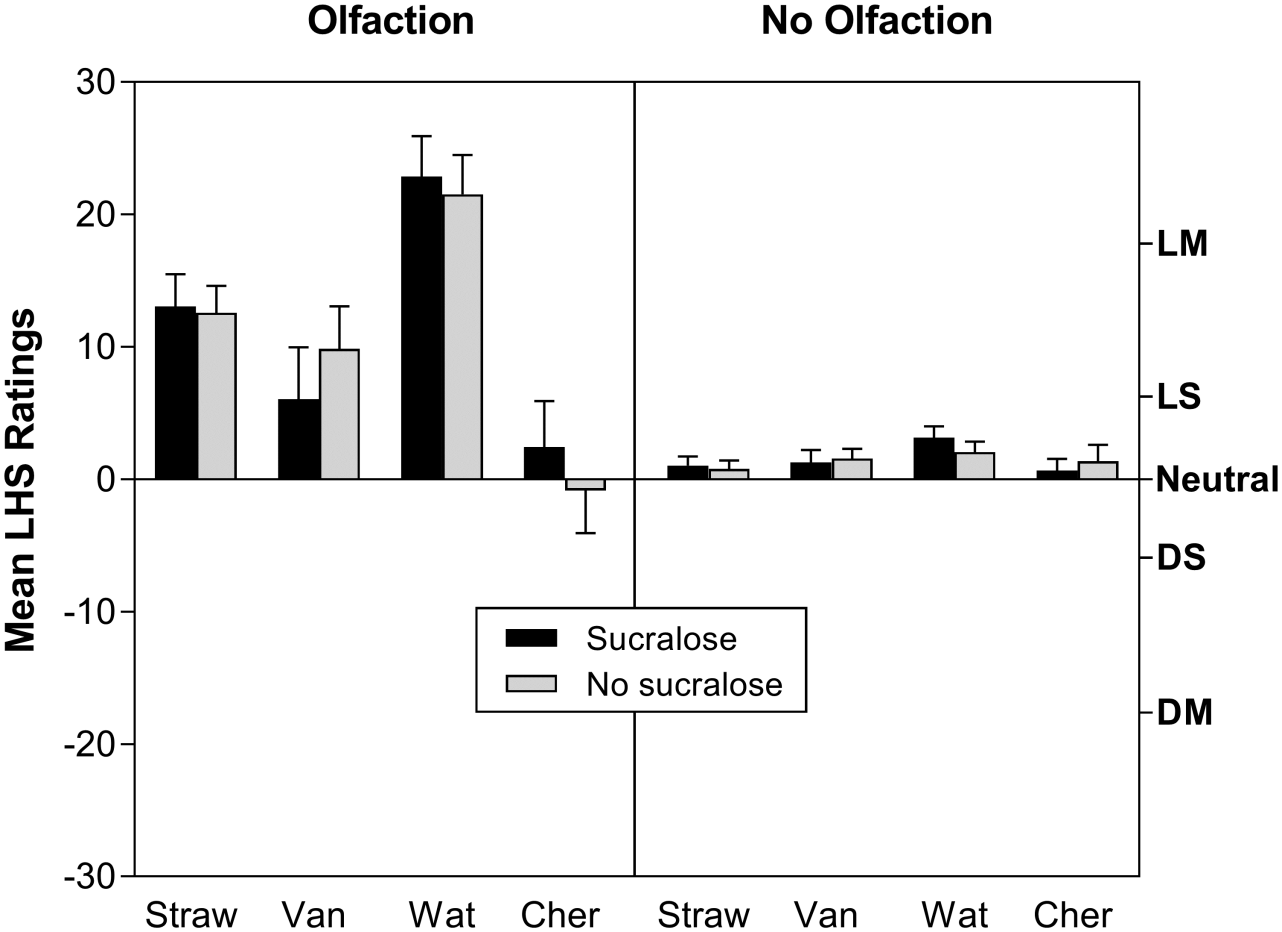
Mean ratings of liking/disliking. Mean ratings of *liking/disliking* across both e-cig devices for all e-liquids sampled alone (gray bars) and with 1% sucralose (black bars). Olfaction = nose open, No Olfaction = nose closed. The data show that liking differed significantly between olfactory conditions, as seen by higher ratings with the nose open, and across flavors. The letters on the right-y axis represent labels on the Labeled Hedonic Scale (LHS): DM = Dislike Moderately, DS = Dislike Slightly, N = Neutral, LS = Like Slightly, LM = Like Moderately. Vertical bars represent standard errors of the mean.

Fig 3 shows the mean ratings of *liking/disliking* for the Olfaction and No Olfaction conditions collapsed across delivery systems. The large increase in *overall flavor* and *sweetness* intensity when the nose was open led to a main effect of Olfaction (p<0.00001), with subjects giving 3 of the 4 flavors higher *liking* ratings in that condition. The low *liking* ratings for the cherry flavor together with the high *liking* ratings for the watermelon flavor contributed to a main effect of e-Liquid (p<0.00001) and a significant 3-way interaction among e-Liquid, Olfaction and Sucralose (p<0.05). There was no significant effect of Delivery System on *liking/disliking*. A trend toward a 3-way interaction among Delivery System, Sucralose, and Olfaction fell just short of statistical significance.

### Analytical chemistry data

Table 1 summarizes the results of the vaping experiments with the data arranged by the type of container used (V2 cartridge or tank) and e-liquid flavor. Additionally, sucralose was quantified in two different locations: in the vapor trapped in the cold-finger trap as well as in the condensate that built up inside the mouthpiece of the respective container over the course of 20 puffs. Statistical analysis showed that there was no influence of flavor on the presented data (all one-way ANOVAs with Bonferroni post-test, p > 0.05) and thus the data across flavors was combined for further analysis.

**Table 1:** Mass change of the e-liquid after 20 puffs as well as amount of condensate found inside of the mouthpiece, the mass, and resulting concentration (μg/mg) of sucralose found in the trapped vapor and the mouthpiece condensate.

	Vapor (found in trap)			Condensate (found in mouthpiece)		
Sample	Mass change of e-liquid reservoir	Total mass of sucralose found in trap	Resulting concentration of sucralose in trap	Amount of condensate found in mouthpiece	Total mass of sucralose found in mouthpiece	Resulting concentration of sucralose in mouthpiece
	(mg)	(μg)	(μg/mg)	(mg)	(μg)	(μg/mg)
**Cartridge**						
Strawberry^ǂ^	102.0 ± 12.5	33.8 ± 8.5	0.33	4.1 ± 1.2	3.38 ± 2.6	0.76
Vanilla^ǂ^	106.9 ± 14.5	21.2 ± 7.1	0.20	3.3 ± 0.5	1.75 ± 1.0	0.51
Watermelon^ǂ^	105.7 ± 18.5	34.7 ± 16.1	0.33	4.3 ± 2.9	3.34 ± 3.0	0.72
Cherry	87.8 ± 9.8	22.0 ± 14.0	0.25	5.0 ± 2.0	2.77 ± 2.2	0.51
**Tank**						
Strawberry	217.0 ± 12.1	45.1 ± 18.1	0.21	14.3 ± 1.3	7.75 ± 3.4	0.54
Vanilla	210.2 ± 22.7	19.2 ± 14.4	0.10	11.2 ± 1.9	4.55 ± 2.0	0.39
Watermelon	202.5 ± 17.5	26.5 ± 14.3	0.13	15.9 ± 5.2	5.62 ± 3.0	0.35
Cherry	198.8 ± 15.5	18.7 ± 2.0	0.09	15.0 ± 4.2	5.50 ± 1.0	0.39

Standard deviation of mass shown. For each sample n = 3, except where marked with ^ǂ^, n = 4.

About twice as much e-liquid was consumed from the tank over the course of 20 puffs compared to the cartridge system (two-tailed t-test, both p < 0.0001) The amount of condensate found inside the mouthpiece of the tank-system was also significantly greater than for the cartridge-system (two-tailed t-test, both p < 0.0001). The latter difference might be explained by the difference in size and geometry of the two mouthpieces. For the cartridge, the channel that delivers the vapor from the vaporizer to the mouth is only ~4mm long with a diameter of ca. 2.5mm, while for the tank the channel is conically shaped with an overall length ~24.4mm and a diameter that increases from ~3.4mm to ~5.2mm at the tip.

**Fig 4:**
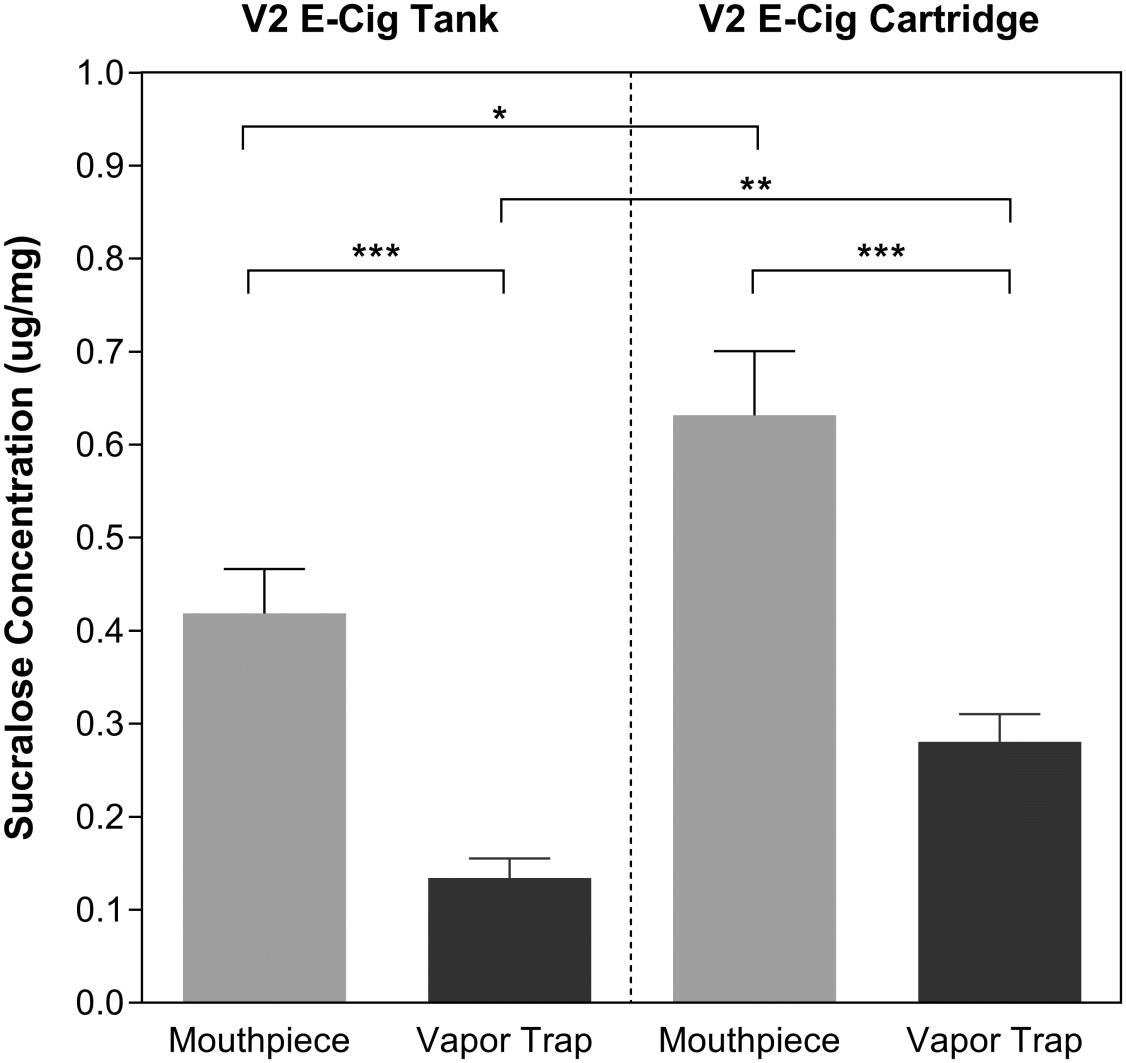
Measured sucralose concentrations. Average sucralose concentration (μg/mg) found across all flavors in the mouthpiece (gray bars) condensate and the vapor trap (black bars). Data from V2 tanks are shown on the left and V2 cartridges shown on the right. Error bars show standard error (n = 12 for tank, n = 15 for cartridge).

Fig 4 shows the average sucralose concentrations found for the cartridge and tank across all 4 flavors. Concentrations were significantly higher in the mouthpiece condensate than in the vapor trap for both the tank and the cartridge (two-tailed t-test, both p< 0.0001), indicating that sucralose tended to accumulate in the mouthpieces. Although the absolute amounts of sucralose in the vapor trap were not significantly different between the cartridge and tank (two-tailed t-test, p = 0.8694; see Table 1), the cartridge yielded higher sucralose concentrations than the tank in both the condensate of the mouthpiece (two-tailed t-test, p< 0.05) and the vapor trap (two-tailed t-test, p< 0.002).

## Discussion

As was pointed out in the Introduction, it is well known that some odors have sweet qualities that can be confused with sweet taste, and that the sweetness of odors sensed retronasally contributes to the perceived sweetness of foods and beverages [18–20]. The present results show that by themselves, sweet odors can produce the characterizing sweetness of e-cigarette sweet and fruity flavors. Given the absence to date of published evidence of artificial sweeteners in commercial e-liquid flavors, it is highly likely that the sweetening agents in most e-cigarette flavors are sweet-smelling odors. In addition, our results show that while adding sucralose to commercial sweet e-liquid flavors can add to their sweetness, the increases in sweetness were proportionally small and were insufficient to significantly increase flavor liking. Although higher concentrations of sucralose could potentially increase sweetness more effectively, the 1% concentration used here is more than 100 times higher than the concentration needed to produce a clearly perceptible sweet taste in aqueous solutions (≈ 0.008%) [35], and informal testing with 5% sucralose did not seem to produce a noticeable increase in e-liquid sweetness.

The finding that sucralose produced significantly more sweetness with the V2 cartridge than with the V2 EX tank system indicates that the effectiveness of adding an artificial sweetener can depend on the e-cigarette delivery system. This result was somewhat surprising because both systems use the same 4.2V battery and 3Ω heating coil and so should produce similar atomizing temperatures. Therefore other design features (see Fig 1) must have affected delivery of sucralose into the vapor. For example, e-liquid delivery to the heating coils is different between the V2 cartridge and the V2 EX tank. Although in both systems the e-liquid is brought to the heating coil via a cotton-fiber blend wicking system, in the cartridge the e-liquid is contained within a sheet of rolled cotton-fiber that surrounds the heated air channel, while in the tank the e-liquid is drawn from the tank into the heating channel through a cotton-fiber strip. These different wicking designs may differentially affect diffusion of sucralose into the heating channel. In addition, as noted above, the mouth piece of the tank system has a much larger surface area on which the aerosol could condense during vaping. During analytical testing we noticed that tiny droplets of condensate often appeared on the outer edge of the cartridge mouth piece but not the tank mouth piece, raising the possibility that sucralose in the condensate could have been tasted if participants touched their tongue tip to the mouth piece. However, because the droplets were observed during analytical testing after bouts of 20 simulated puffs, it is unclear whether they would form after the 3 puffs that were used during psychophysical testing.

As shown in Table 1, the greater total mass of sucralose measured in the mouthpiece and trap for the tank compared to the cartridge system seems in conflict with the higher ratings of overall flavor and sweetness when sucralose was added in the cartridge system. However, it is well known that the potency of taste stimulation depends more upon stimulus concentration than upon stimulus mass, as is evident from the use of molarity or molar concentration (defined as the number of moles of a substance per liter of solute) as the standard measure of stimulus strength in taste research [36]. The significantly higher sucralose concentration in the vapor of the cartridge compared to the vapor from the tank is therefore a likely reason for the higher sweetness reported with the cartridge (Table 1 and Fig 4). The source of this difference in sucralose concentration is unclear, as it could depend upon one or more of the differences between the delivery systems discussed above. The finding nevertheless demonstrates that the effectiveness of adding non-volatile sweeteners to e-liquids can depend in part on e-cigarette design, and so may vary even more between delivery systems that also have different atomizer voltages and temperatures.

More generally, the evidence that the e-cigarette delivery system can alter the effectiveness of sucralose as a sweetening agent has implications for the use of high-potency sweeteners in other types of tobacco products. While non-volatile sweeteners like sucralose have the potential to strongly affect the flavor of products in which sweeteners come in direct contact with the mouth and tongue (e.g., chewing tobacco or cigars in which sweeteners are added to the tobacco leaf wrapping), the present findings suggest their effectiveness in products that rely on delivery of flavoring agents solely via inhalation (e.g., hookas) is likely to be relatively small compared to volatile flavor molecules and dependent upon the specific design of the devices.

## Conclusions

The present results demonstrate that the perceived sweetness of flavors derives primarily from volatile components of e-liquids that are sensed via retronasal olfaction rather than by taste. Although it was found that the artificial sweetener sucralose can add to the sweetness of flavors, the amount of sweetness added was small and had little effect on flavor liking. However, the added sweetness depended on the e-cigarette delivery system. Together these findings suggest that any future regulation of flavor sweetness in e-cigarettes should focus primarily on the volatile (olfactory) constituents of e-liquids, with the caveat that use of more powerful e-cigarette systems, or systems that are specifically designed to deliver artificial sweeteners into the inhaled aerosol, may enable non-volatile sweeteners to contribute more strongly to the appeal of e-cigarettes.

## References

[1] Berg CJ. Preferred flavors and reasons for e-cigarette use and discontinued use among never, current, and former smokers. Int J Public Health. 2016;61(2):225–36. doi: 10.1007/s00038-015-0764-x 26582009 10.1007/s00038-015-0764-x PMC4808473

[2] Grana RA, Ling PM. "Smoking revolution": a content analysis of electronic cigarette retail websites. Am J Prev Med. 2014;46(4):395–403. doi: 10.1016/j.amepre.2013.12.010 24650842 10.1016/j.amepre.2013.12.010 PMC3989286

[3] Farsalinos KE, Romagna G, Tsiapras D, Kyrzopoulos S, Spyrou A, Voudris V. Impact of flavour variability on electronic cigarette use experience: an internet survey. Int J Environ Res Public Health. 2013;10(12):7272–82. doi: 10.3390/ijerph10127272 24351746 10.3390/ijerph10127272 PMC3881166

[4] Harrell MB, Weaver SR, Loukas A, Creamer M, Marti CN, Jackson CD, et al Flavored e-cigarette use: Characterizing youth, young adult, and adult users. Prev Med Rep. 2017;5:33–40. doi: 10.1016/j.pmedr.2016.11.001 27896041 10.1016/j.pmedr.2016.11.001 PMC5121224

[5] Krishnan-Sarin S, Morean ME, Camenga DR, Cavallo DA, Kong G. E-cigarette Use Among High School and Middle School Adolescents in Connecticut. Nicotine Tob Res. 2015;17(7):810–8. doi: 10.1093/ntr/ntu243 25385873 10.1093/ntr/ntu243 PMC4674435

[6] Ford A, MacKintosh AM, Bauld L, Moodie C, Hastings G. Adolescents' responses to the promotion and flavouring of e-cigarettes. Int J Public Health. 2016;61(2):215–24. doi: 10.1007/s00038-015-0769-5 26650455 10.1007/s00038-015-0769-5 PMC4819499

[7] Bonhomme MG, Holder-Hayes E, Ambrose BK, Tworek C, Feirman SP, King BA, et al Flavoured non-cigarette tobacco product use among US adults: 2013–2014. Tob Control. 2016;25(Suppl 2):ii4–ii13. doi: 10.1136/tobaccocontrol-2016-053373 . 27794065 10.1136/tobaccocontrol-2016-053373 PMC5515238

[8] Goldenson NI, Kirkpatrick MG, Barrington-Trimis JL, Pang RD, McBeth JF, Pentz MA, et al Effects of sweet flavorings and nicotine on the appeal and sensory properties of e-cigarettes among young adult vapers: Application of a novel methodology. Drug Alcohol Depend. 2016;168:176–80. doi: 10.1016/j.drugalcdep.2016.09.014 27676583 10.1016/j.drugalcdep.2016.09.014 PMC5086287

[9] Kim H, Lim J, Buehler SS, Brinkman MC, Johnson NM, Wilson L, et al Role of sweet and other flavours in liking and disliking of electronic cigarettes. Tob Control. 2016;25(Suppl 2):ii55–ii61. doi: 10.1136/tobaccocontrol-2016-053221 . 27708124 10.1136/tobaccocontrol-2016-053221 PMC5489117

[10] Bold KW, Kong G, Cavallo DA, Camenga DR, Krishnan-Sarin S. E-cigarette susceptibility as a predictor of youth initiation of e-cigarettes. Nicotine Tob Res. 2016 doi: 10.1093/ntr/ntw393 . 28035000 10.1093/ntr/ntw393 PMC5868212

[11] Kosmider L, Sobczak A, Prokopowicz A, Kurek J, Zaciera M, Knysak J, et al Cherry-flavoured electronic cigarettes expose users to the inhalation irritant, benzaldehyde. Thorax. 2016;71(4):376–7. doi: 10.1136/thoraxjnl-2015-207895 26822067 10.1136/thoraxjnl-2015-207895 PMC4937616

[12] Miao S, Beach ES, Sommer TJ, Zimmerman JB, Jordt SE. High-Intensity Sweeteners in Alternative Tobacco Products. Nicotine Tob Res. 2016;18(11):2169–73. doi: 10.1093/ntr/ntw141 27217475 10.1093/ntr/ntw141 PMC5055742

[13] Sherwood CL, Boitano S. Airway epithelial cell exposure to distinct e-cigarette liquid flavorings reveals toxicity thresholds and activation of CFTR by the chocolate flavoring 2,5-dimethypyrazine. Resp Res. 2016;17 doi: 10.1186/s12931-016-0369-9 27184162 10.1186/s12931-016-0369-9 PMC4869201

[14] Nelson G, Hoon MA, Chandrashekar J, Zhang Y, Ryba NJ, Zuker CS. Mammalian sweet taste receptors. Cell. 2001;106(3):381–90. . 11509186 10.1016/s0092-8674(01)00451-2

[15] Fernstrom JD, Munger SD, Sclafani A, de Araujo IE, Roberts A, Molinary S. Mechanisms for sweetness. J Nutr. 2012;142(6):1134S–41S. doi: 10.3945/jn.111.149567 22573784 10.3945/jn.111.149567 PMC3738222

[16] Yeomans MR, Mobini S, Elliman TD, Walker HC, Stevenson RJ. Hedonic and sensory characteristics of odors conditioned by pairing with tastants in humans. J Exp Psychol Anim Behav Process. 2006;32(3):215–28. doi: 10.1037/0097-7403.32.3.215 . 16834490 10.1037/0097-7403.32.3.215

[17] Stevenson RJ, Prescott J, Boakes RA. The Acquisition of Taste Properties by Odors. Learning and Motivation. 1995;26(4):433–55. doi: 10.1016/S0023-9690(05)80006-2

[18] Tieman D, Bliss P, McIntyre LM, Blandon-Ubeda A, Bies D, Odabasi AZ, et al The chemical interactions underlying tomato flavor preferences. Curr Biol. 2012;22(11):1035–9. doi: 10.1016/j.cub.2012.04.016 . 22633806 10.1016/j.cub.2012.04.016

[19] Small DM, Prescott J. Odor/taste integration and the perception of flavor. Exp Brain Res. 2005;166(3–4):345–57. doi: 10.1007/s00221-005-2376-9 . 16028032 10.1007/s00221-005-2376-9

[20] Auvray M, Spence C. The multisensory perception of flavor. Conscious Cogn. 2008;17(3):1016–31. doi: 10.1016/j.concog.2007.06.005 17689100 10.1016/j.concog.2007.06.005

[21] Spence C. Oral referral: On the mislocalization of odours to the mouth. Food Quality and Preference. 2016;50:117–28. http://dx.doi.org/10.1016/j.foodqual.2016.02.006.

[22] Green BG, Nachtigal D, Hammond S, Lim J. Enhancement of retronasal odors by taste. Chem Senses. 2012;37(1):77–86. doi: 10.1093/chemse/bjr068 21798851 10.1093/chemse/bjr068 PMC3243899

[23] Frank RA, Vanderklaauw NJ, Schifferstein HNJ. Both Perceptual and Conceptual Factors Influence Taste-Odor and Taste-Taste Interactions. Percept Psychophys. 1993;54(3):343–54. doi: 10.3758/Bf03205269 8414893 10.3758/bf03205269

[24] Lim J, Padmanabhan A. Retronasal olfaction in vegetable liking and disliking. Chem Senses. 2013;38(1):45–55. doi: 10.1093/chemse/bjs080 . 23001321 10.1093/chemse/bjs080

[25] Tierney PA, Karpinski CD, Brown JE, Luo W, Pankow JF. Flavour chemicals in electronic cigarette fluids. Tob Control. 2016;25(e1):e10–5. doi: 10.1136/tobaccocontrol-2014-052175 25877377 10.1136/tobaccocontrol-2014-052175 PMC4853541

[26] Lisko JG, Tran H, Stanfill SB, Blount BC, Watson CH. Chemical Composition and Evaluation of Nicotine, Tobacco Alkaloids, pH, and Selected Flavors in E-Cigarette Cartridges and Refill Solutions. Nicotine Tob Res. 2015;17(10):1270–8. doi: 10.1093/ntr/ntu279 25636907 10.1093/ntr/ntu279 PMC4573955

[27] Verification SSoB. Biochemical verification of tobacco use and cessation. Nicotine Tob Res. 2002;4(2):149–59. doi: 10.1080/14622200210123581 . 12028847 10.1080/14622200210123581

[28] Green BG, Shaffer GS. The sensory response to capsaicin during repeated topical exposures: Differential effects on sensations of itching and pungency. Pain. 1993;53:323–34. 8351161 10.1016/0304-3959(93)90228-H

[29] Green BG, Dalton P, Cowart BJ, Shaffer GS, Rankin KM, Higgins J. Evaluating the 'Labeled Magnitude Scale' for measuring sensations of taste and smell. Chem Senses. 1996;21:323–34. 8670711 10.1093/chemse/21.3.323

[30] Bartoshuk LM, Duffy VB, Green BG, Hoffman HJ, Ko CW, Lucchina LA, et al Valid across-group comparisons with labeled scales: the gLMS versus magnitude matching. Physiol Behav. 2004;82(1):109–14. doi: 10.1016/j.physbeh.2004.02.033 . 15234598 10.1016/j.physbeh.2004.02.033

[31] Lim J, Wood A, Green BG. Derivation and evaluation of a labeled hedonic scale. Chem Senses. 2009;34(9):739–51. doi: 10.1093/chemse/bjp054 19833660 10.1093/chemse/bjp054 PMC2762053

[32] Green BG, Shaffer GS, Gilmore MM. Derivation and Evaluation of a Semantic Scale of Oral Sensation Magnitude with Apparent Ratio Properties. Chemical Senses. 1993;18(6):683–702. doi: 10.1093/chemse/18.6.683

[33] Talih S, Balhas Z, Eissenberg T, Salman R, Karaoghlanian N, El Hellani A, et al Effects of user puff topography, device voltage, and liquid nicotine concentration on electronic cigarette nicotine yield: measurements and model predictions. Nicotine Tob Res. 2015;17(2):150–7. doi: 10.1093/ntr/ntu174 25187061 10.1093/ntr/ntu174 PMC4837998

[34] Soh L, Connors KA, Brooks BW, Zimmerman J. Fate of sucralose through environmental and water treatment processes and impact on plant indicator species. Environ Sci Technol. 2011;45(4):1363–9. doi: 10.1021/es102719d . 21235203 10.1021/es102719d

[35] Schiffman SS, Sattely-Miller EA, Graham BG, Zervakis J, Butchko HH, Stargel WW. Effect of repeated presentation on sweetness intensity of binary and ternary mixtures of sweeteners. Chem Senses. 2003;28(3):219–29. 12714444 10.1093/chemse/28.3.219

[36] Pfaffmann C, Young PT, Dethier VG, Richter CP, Stellar E. The preparation of solutions for research in chemoreception and food acceptance. J Comp Physiol Psychol. 1954;47(1):93–6. . 13130740 10.1037/h0053778

